# *In Vitro* Palmitate Treatment of Myotubes from Postmenopausal Women Leads to Ceramide Accumulation, Inflammation and Affected Insulin Signaling

**DOI:** 10.1371/journal.pone.0101555

**Published:** 2014-07-07

**Authors:** Julie Abildgaard, Darren C. Henstridge, Anette T. Pedersen, Katherine G. Langley, Camilla Scheele, Bente Klarlund Pedersen, Birgitte Lindegaard

**Affiliations:** 1 The Centre of Inflammation and Metabolism and Centre for Physical Activity Research, Department of Infectious Diseases and CMRC, Rigshospitalet, Copenhagen, Denmark; 2 Cellular and Molecular Metabolism Laboratory, Baker IDI Heart and Diabetes Institute, Melbourne, Australia; 3 Department of Gynaecology, Rigshospitalet, Copenhagen, Denmark; Pennington Biomed Research Center, United States of America

## Abstract

Menopause is associated with an increased incidence of insulin resistance and metabolic diseases. In a chronic palmitate treatment model, we investigated the role of skeletal muscle fatty acid exposure in relation to the metabolic deterioration observed with menopause. Human skeletal muscle satellite cells were isolated from premenopausal (n = 6) and postmenopausal (n = 5) women. In an *in vitro* model, the myotubes were treated with palmitate (300 µM) for one-, two- or three days during differentiation. Effects on lipid accumulation, inflammation and insulin signaling were studied. Palmitate treatment led to a 108% (CI 95%: 50%; 267%) increase in intramyocellular ceramide in the myotubes from the postmenopausal women (post-myotubes) compared with a 26% (CI 95%: −57%; 96%) increase in myotubes from the premenopausal women (pre-myotubes), (p<0.05). Furthermore, post-myotubes had a 22% (CI 95%: 4%; 34%) increase in pJNK (p = 0.04) and a 114% (CI 95%: 50%; 177%) increase in Hsp70 protein expression (p = 0.03) after three days of palmitate treatment, compared with pre-myotubes, in which no increase in either pJNK (−12% (CI 95: −26%; 2%)) or Hsp70 (7% (CI 95: −78%; 91%)) was detected. Furthermore, post-myotubes showed a blunted insulin stimulated phosphorylation of AS160 in response to chronic palmitate treatment compared with pre-myotubes (p = 0.02). The increased intramyocellular ceramide content in the post-myotubes was associated with a significantly higher mRNA expression of Serine Palmitoyltransferase1 (SPT1) after one day of palmitate treatment (p = 0.03) in post-myotubes compared with pre-myotubes. Our findings indicate that post-myotubes are more prone to develop lipid accumulation and defective insulin signaling following chronic saturated fatty acid exposure as compared to pre-myotubes.

## Introduction

At the time of menopausal transition women experience a significant increase in the incidence of metabolic diseases including metabolic syndrome [1], [2], diabetes [3] and cardiovascular disease [4]–[6]. The increased incidence of metabolic diseases during menopause is associated with changes in body composition, as postmenopausal women have decreased skeletal muscle mass [7]–[9] as well as increased visceral fat mass [9], [10]. Furthermore, postmenopausal women have lower whole body fat oxidation rates compared to premenopausal women [9], [10], a phenomenon known to lead to obesity [11] and dyslipidemia [12]. As a consequence of inadequate fat oxidation, excess adiposity often leads to ectopic fat storage of lipid metabolites including triacylglycerols (TAGs), diacylglycerols (DAGs) and ceramides in metabolic tissues such as skeletal muscle. These stored metabolites could be a contributing factor to the increasing insulin resistance observed after the menopausal transition.

Insulin resistance in skeletal muscle following excessive lipid load is thought to be caused by an accumulation of toxic lipid metabolites, including ceramides, which increase inflammation in the skeletal muscle [13]–[15]. Ceramides can be formed either by *de novo* synthesis or through catabolism of sphingomyelin, a phospholipid component of the cell membrane [16], [17]. *De novo* ceramide synthesis has been found to play an important role in ceramide accumulation in response to a lipid overload [18]. The rate-limiting step in the *de novo* ceramide synthesis is performed by the enzyme Serine C-palmitoyltransferase (SPT). Among other mechanisms, ceramides operate as second messengers by altering the activity of kinases, phosphatases or transcription factors [19], . Impaired insulin signaling is associated with ceramide accumulation and has been shown to be a consequence of the ability of ceramides to phosphorylate and thereby activate the stress kinase JNK [13], [14] as well as decreasing the phosphorylation of Akt [15]. The roles of TAGs and DAGs in the development of insulin resistance are more controversial [18], [21], [22].

In animal models, loss of ovarian function leads to increased levels of intramyocellular lipids [23]. Furthermore, estrogen deprivation combined with a high lipid load has been found to lead to an even more pronounced skeletal muscle insulin resistance associated with both decreased phosphorylation of Akt and increased phosphorylation of JNK [24]. Thus, it is possible that the insulin resistance observed in postmenopausal women is partly due to an accumulation of lipid metabolites; however, to the best of our knowledge, no earlier studies have investigated this matter.

Excessive fatty acid accumulation in skeletal muscle cells may also lead to oxidative stress, with accumulation of reactive oxygen species, ultimately resulting in insulin resistance [25]. Hsp70 is a heat shock protein that is increased in response to heat stress and toxic compounds [26], [27] and has been shown to prevent lipid-induced insulin resistance [28].

Skeletal muscle is critical in whole body metabolism as it plays a major role in whole body insulin sensitivity [29] and is responsible for up to one third of the oxygen consumption at rest [30]. Thus, changes in skeletal muscle metabolism are often associated with the development of metabolic diseases. A number of factors influence insulin sensitivity in skeletal muscle including increased levels of circulating lipids [31], and accumulation of reactive oxygen species [32], cytokines, e.g. interleukins, and stress hormones [33]. As menopause is associated with the development of dyslipidemia [8]–[10], it is possible that an inability of postmenopausal skeletal muscle to oxidize the excess lipid greatly contributes to the development of insulin resistance after menopause.

In the present study, we tested the hypothesis that myotubes from postmenopausal women (post-myotubes) develop lipid accumulation and inflammation (increases in p-JNK and Hsp70 protein expression) in response to a chronic lipid load, to a higher extent than myotubes from premenopausal women (pre-myotubes). Furthermore, we sought to investigate if these changes were associated with differences between groups in lipid metabolism and insulin sensitivity.

## Methods

### Ethics statement

Participants were provided with both oral and written information about the experimental procedures before giving their written, informed consent. The study was approved by the Ethical Committee of Copenhagen (H-3-2010-073), Denmark and performed according to the declaration of Helsinki.

### Subjects

The subjects were randomly picked from a larger cohort of pre- and postmenopausal women [9], and were matched by age, BMI and VO2max. Exclusion criteria were: 1) infections during the last four weeks, 2) chronic disease such as diabetes and other metabolic disorders, 3) use of medication including hormone therapy, 4) smoking, 5) hysterectomy and/or oophorectomy, 6) premature ovarian failure, 7) BMI>30 kg/m2.

Six pre- and six postmenopausal women were enrolled in the study. However, because of fibroblast contamination in the satellite cells from one of the postmenopausal women, this subject was excluded. The women were defined as pre- and postmenopausal based on the following criteria: Premenopausal (menstrual bleeding within the last 12 months and FSH<20 IU/l); Postmenopausal (amenorrhea for more than 12 months and FSH>20 IU/l). Premenopausal women with a menstrual period within the last three months were enrolled in the follicular phase of the first coming menstrual period (on days three - seven of their menstrual cycle). The postmenopausal women were enrolled on a random day. Study group characteristics are presented in *table 1*.

**Table 1:** Subject characteristics.

	Premenopausal	Postmenopausal
*n*	*6*	*5*
Age (*years*)	51.2±0.65	52.6±0.19
BMI	23.2±0.71	22.9±1.06
VO2max (*ml/kg*)	33.10±2.22	31.40±2.09
Lean body mass (*kg*)	41.93±1.43	39.51±1.41
Fat mass (*kg*)	19.77±1.42	20.62±1.64
FSH (*iu/l*)	14.38±2.91	74.74±11.70**

Data are presented as means ± SEM.

** Significantly different from premenopausal, p<0.001.

The subjects were instructed not to perform any vigorous exercise 24 hours prior to the experiments. They reported to the laboratory between 7 and 9 am after an overnight fast. Skeletal muscle biopsies were obtained from the vastus lateralis of the quadriceps muscle with a modified Bergström needle (including suction) under local anesthesia with two % lidocaine.

### Satellite cell isolation, proliferation and differentiation

Muscle satellite cells were isolated from the vastus lateralis of the quadriceps muscle as previously described [34]. After removal of fat and connective tissue, the biopsy was digested in a 10 mL buffer containing trypsin and collagenase II for 15 min. To minimize fibroblast contamination, cells were preseeded in a culture dish for three h in F10/HAM, 20% FBS, 1% PS, and 1% Fungizone (all from Invitrogen, Taastrup, Denmark). Unattached cells were then removed and seeded into a culture flask, precoated with Matrigel (BD Biosciences, San Jose, CA, US). After 4 days of incubation, the cell culture medium was changed and subsequently every second day thereafter. Cell cultures were expanded and then seeded on Matrigel-coated plates for differentiation. Next, 100% confluent cells were incubated with DMEM (Invitrogen, Taastrup, Denmark) containing 1 g/L glucose, 10% FBS, and 1% P/S to allow cells to align; after two days, the medium was changed to DMEM containing 1 g/L glucose, 2% HS, and 1% P/S. Culture medium was changed to serum-free DMEM containing 1 g/L glucose two h prior to treatment with Insulin. For all experiments, cells were used at day seven of differentiation at passage four-six. Palmitate treatment was given the last one-, two- and three days of differentiation as previously described [35].

### Palmitate treatment

The media consisted of DMEM containing 1 g/L glucose, 2% HS, and 1% P/S, 0.5% human serum albumin either conjugated to 300 *µ*M palmitate or the equivalent volume of 100% ethanol. Media were refreshed every 24 h for these last three days of differentiation. Pictures were taken of each cell ID before harvest to ensure that there were no visual differences in myotube differentiation and density as a consequence of palmitate treatment or menopausal status.

Myosin heavy chain (MyHC), a component of the motor protein myosin in the skeletal muscle, was measured by western blot to assess myotube differentiation.

We aimed to create a for the muscle cells metabolically unfavorable environment, known to affect inflammation and insulin signaling [25]. A lipid mixture of palmitate and oleate would have been more physiological, however, oleate has been found to reverse the metabolic deterioration that palmitate is known to cause [25]. Thus, the present model of palmitate treatment allowed us to study the impact on skeletal muscle cells unaffected by other lipids that could mask potential findings.

### Cell lysis

Cells were rinsed once in ice-cold PBS and lysed in 20 mmol/L Tris, pH 7.5, 150 mmol/L NaCl, 1 mmol/L EGTA, 1 mmol/L EDTA, 0.1% Triton X-100, Complete mini protease inhibitor (Boehringer-Roche Diagnostics, Copenhagen, Denmark) (1 tablet/10 mL), and 1% phosphatase inhibitor cocktail (Sigma-Aldrich, Brøndby, Denmark). Whole cell lysates were centrifuged (12,000 g at 4°C for 10 min), and supernatants were removed for storage at −80°C until required. Protein concentration in lysates was measured by the Bradford reagent (BioRad, Hercules, CA, USA) [36].

### Western blotting

Twenty micrograms of whole cell lysates were subjected to SDS-PAGE using BioRad 4–15% precast gels and wet transfer. Polyvinylidene difluoride (PVDF) membranes were probed with primary antibodies raised against the protein of interest; p-ACC(Ser79) (#3661, Cell Signaling Technology, Danvers, MA, USA), p-JNK(Thr183/Tyr185) (#9251, Cell Signaling Technology, Danvers, MA, USA), Hsp70 (#4872, Cell Signaling Technology, Danvers, MA, USA), p-Akt(Ser473) (#9271, Cell Signaling Technology, Danvers, MA, USA), CD36 (FAB19551A, R&D systems, Minneapolis, MN, USA), MyHC (Iowa Hybridoma Bank, Iowa City, IA, USA), p-GSK3 α/β(Ser21/9) (#9331 Cell Signaling Technology, Danvers, MA, USA), p-AS160(Thr642) (#4288 Cell Signaling Technology, Danvers, MA, USA). Detection of primary antibodies was performed using appropriate peroxidase-conjugated IgG and protein signals visualized using FEMTO enhanced chemiluminescence and BioRad Chemidoc XRS imager. Total protein content was quantified using reactive brown 10 (Sigma-Aldrich, St-Louis, MO, USA). Quantification of immunoblots was performed using ImageJ (NIH, Bethesda, MD, USA http://rsb.info.nih.gov/ij).

### RNA-isolation and quantitative real-time PCR

Total RNA was extracted from cells using TriZol (Invitrogen, Carlsbad, CA, USA) according to the manufacturer's protocol. RNA was resuspended in 30 µl of Nuclease free water. The concentration of the isolated RNA and the ratio of absorbance at 260∶280 nm were measured using a spectrophotometer (BMG Labtechnologies, Offenburg, Germany). RNA was reverse transcribed using random hexamers employing a high capacity reverse transcription kit (Applied Biosystems, Foster City, CA, USA) according to the manufacturer's protocol. The RNA levels of analysed genes and the endogenous control 18S were determined by quantitative real time PCR using a ViiA 7 sequence detector (Applied Biosystems, Foster City, CA, USA). Primers and MGB probes were designed using Primer Express software (Applied Biosystems, Foster City, CA, USA) or obtained using the Universal Probe Library (Roche Applied Science, Indianapolis, IN, USA). Primers and probes were premixed with Master Mix (Applied Biosystems, Foster City, CA, USA) and distributed into 384-well MicroAmp optical plates (Applied Biosystems, Foster City, CA, USA). A twofold dilution series was made from a pooled sample. This was run on each plate together with the samples and used to construct a standard curve from which the mRNA content of the target genes was calculated in triplicates, using the standard curve method.

The CPTI isoform used in this study was CPTIB, which is expressed in skeletal muscle cells.

### Lipid metabolite analyses

TAG, DAG and ceramide content were determined in the myotube cell lysates using methods previously described [37]. Briefly, lipid metabolite analyses were performed on cell lysates that were homogenised in 100 µl PBS buffer, pH 7.47. Lipids were extracted from 10 µg protein in 96-well plates, whereby there was no partitioning between the aqueous and organic phases, recovering all lipids in a single phase suitable for liquid chromatography–mass spectrometry analysis. Lipid analyses (including analysis of TAGs, DAGs and ceramides) were performed by liquid chromatography, electrospray ionisation–tandem mass spectrometry using an HP 1200 liquid chromatography system combined with a PE Sciex API 4000 Q/TRAP mass spectrometer with a turbo-ionspray source (350°C) and Analyst 1.5 data system as previously described [37]. TAGs, DAGs and ceramides were all measured from the same lipid extracted sample. The specific lipid species differing by mass were subsequently identified via mass spectrometry analysis and quantified. The TAG, DAG and ceramide levels of all lipid species were summed to give the total pool for each lipid class. As an example ceramide data is displayed as total ceramides and is the sum of cer 16:0, cer 18:0, cer 20:0, cer 22:0, cer 24:0 and cer 24:1 species.

### Statistical analyses

All analyses were performed using SAS 9.2. All data were normalized to the premenopausal control group. P<0.05 was considered as significant. Data were analysed with PROC MIXED with group (premeopausal postmenopausal) and palmitate stimulation (none, one, two, or three days of stimulation) as fixed variables and a random subject-specific component was introduced on the baseline level that allowed adjusting for the inter-individual variations. If an interaction between the two variable revealed significant differences, a Bonferroni post hoc test was used to locate the specific differences (n = 7). When appropriate, values (age, FSH, p-JNK, Hsp70, TAGs, DAGs, ceramides, pAS160) were logarithmically transformed to ensure normality and equal variance. Pearson's correlations were used to examine the relationship between intramyocellular lipids (TAGs, DAGs and ceramides) and SPT1, p-JNK or Hsp70. Data are presented as means ± SEM.

## Results

### Study group characteristics

The two groups were not significantly different in regard to age, BMI, VO2 max and body composition, *table 1*. The postmenopausal women had significantly higher levels of circulating follicle stimulating hormone (FSH) compared to the premenopausal women (p = 0.0004), *table 1*.

### Lipid metabolite accumulation following palmitate treatment

**Figure 1:**
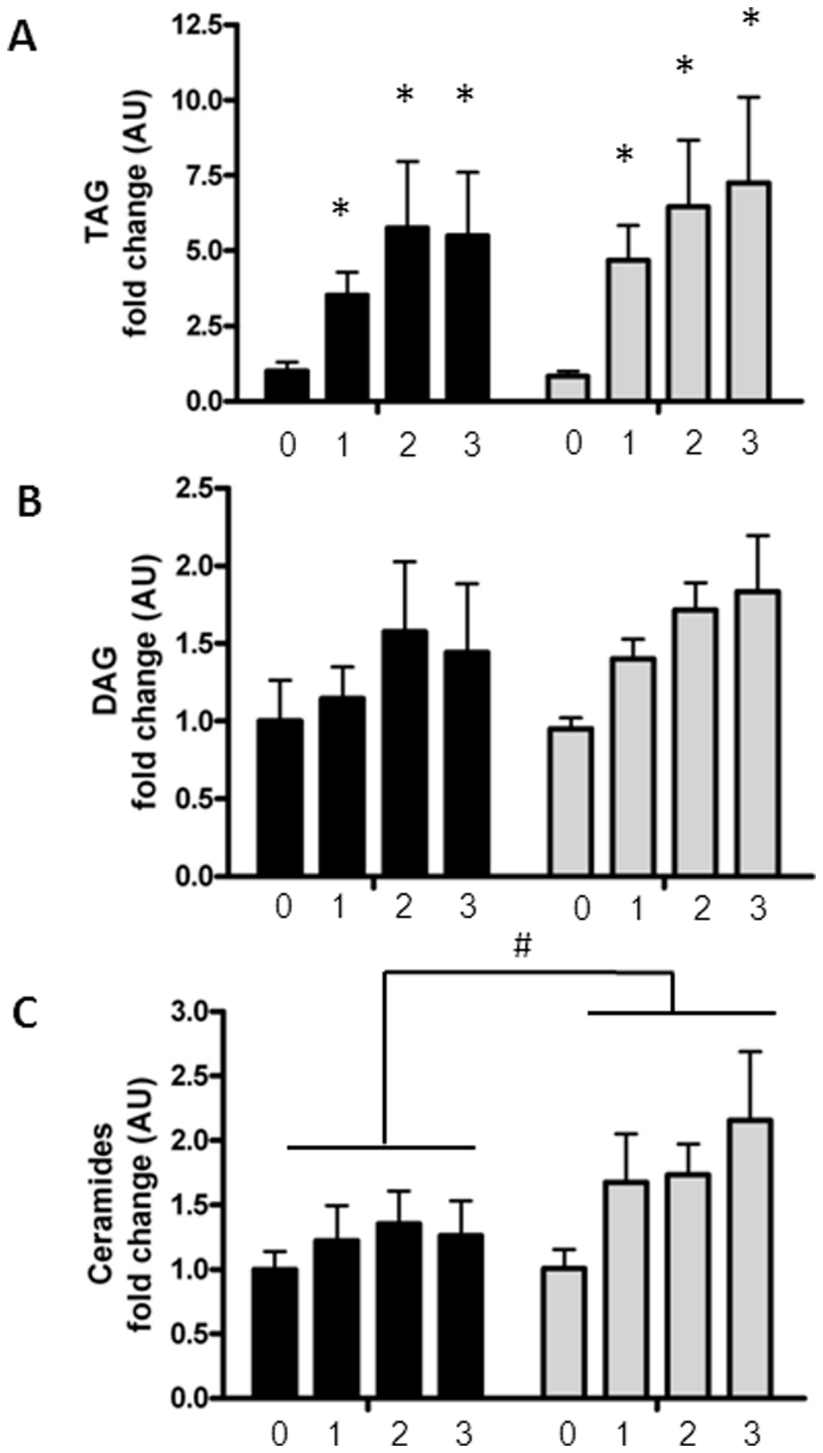
Lipid metabolite content in palmitate treated myotubes from premenopausal and postmenopausal women. Satellite cells were isolated from vastus lateralis biopsies from premenopausal and postmenopausal women. Cells were grown in culture until mature myotubes were formed. Myotubes were treated with palmitate (300 µM) in the last one, two or three days of differentiation. Triacylglycerol (TAG), diacylglycerol (DAG) and ceramide content were determined in the myotube cell lysates by liquid chromatography. Content of lipid metabolites in pre-myotubes (n = 6, black bars) and post-myotubes (n = 5, white bars) in response to palmitate treatment, (A) Intramyocellular TAG levels increased in response to palmitate treatment in both pre- and post-myotubes, (B) Intramyocellular DAG levels were unaffected by both palmitate treatment and menopausal status, (C) Intramyocellular ceramide levels were higher in post-myotubes compared to pre-myotubes. Significant effect of palmitate treatment; * p<0.05. Significantly different from premenopausal; # p<0.05. 0: Zero days of palmitate treatment (control), 1: One day of palmitate treatment, 2: Two days of palmitate treatment, 3: Three days of palmitate treatment. All data were normalized to the premenopausal control group. Data are presented as means ± SEM.

Palmitate treatment led to a significant increase in intracellular TAGs in both pre- and post-myotubes (p<0.0001), whereas there was no increase in the content of diacylglycerols (DAGs), *figure 1*.

Palmitate treatment led to a greater ceramide accumulation in post-myotubes compared to pre-myotubes (108% (CI 95%: 50%; 267%) vs. 26% (CI 95%: −57%; 196%), (p<0.05)). There was no difference in basal levels of ceramide in the myotubes (premenopausal 708.81 pmol/mg protein ± 81.70 vs. postmenopausal 714.63 pmol/mg protein ±123.79).

The increased intramyocellular ceramide content was primarily driven by an increase in Cer16:0, which was responsible for 61% of the total ceramide content after 3 days of palmitate treatment. However, all subspecies of ceramide were significantly increased by palmitate treatment, except from Cer24:1 (data not shown),

### Inflammatory and cell stress response to palmitate stimulation

We then sought to clarify if the increased accumulation of ceramides in the post-myotubes was associated with increased inflammation. In the basal states, none of the inflammatory parameters (p-JNK and Hsp70 protein expression) differed between pre- and post-myotubes.

**Figure 2:**
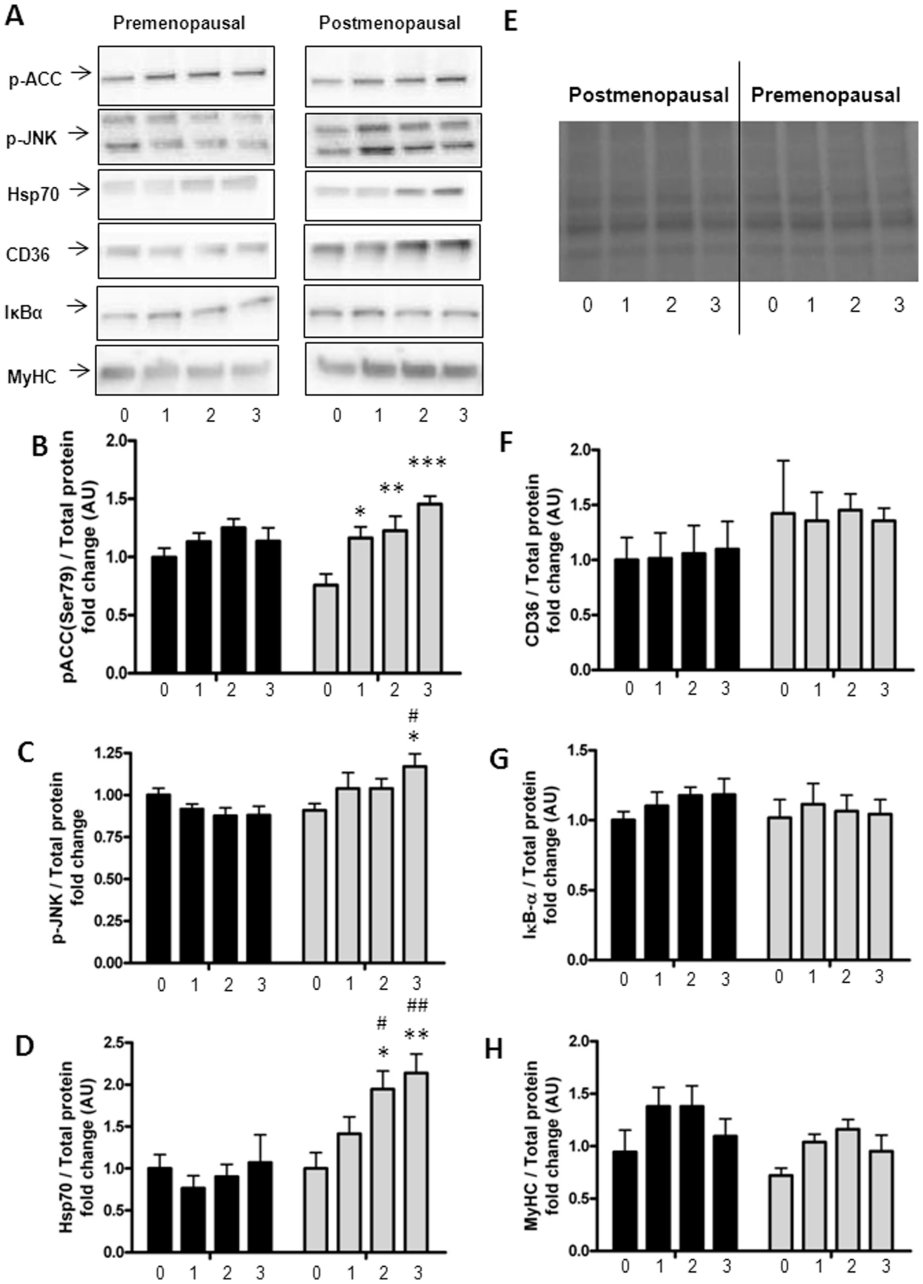
Protein expression in palmitate treated myotubes from premenopausal and postmenopausal women. Satellite cells were isolated from vastus lateralis biopsies from premenopausal and postmenopausal women. Cells were grown in culture until mature myotubes were formed. Myotubes were treated with palmitate in the last one, two or three days of differentiation. Protein lysates from the myotubes were immunoblottet to assess the protein expression of specific proteins as stated below. Protein expression in pre-myotubes (n = 6, black bars) and post-myotubes (n = 5, white bars) in response to palmitate treatment, (A) Representative immunoblots of all investigated proteins, (B) p-ACC-Ser79 (280 kDa) was increased only in post-myotubes in response to palmitate treatment, (C) p-JNK-Thr183/Tyr185 (54/46 kDa) was increased after three days of palmitate treatment in post-myotubes and reached significantly higher levels than in post-myotubes, (D) Hsp70 (72 kDa) was increased after two and three days of palmitate treatment in post-myotubes, and reached significantly higher levels than in pre-myotubes, (E) Representative blot of total protein, (F) CD36 (88 kDa) was unaffected by both palmitate treatment and menopausal status, (G) IκBα (39 kDa) was unaffected by both palmitate treatment and menopausal status, (H) MyHC (230 kDa) was unaffected by both palmitate treatment and menopausal status. Significantly different from control; * p<0.05, ** p<0.01, *** p<0.001. Significantly different from premenopausal, at same palmitate exposure time; # p<0.05, ## p<0.01. 0: Zero days of palmitate treatment (control), 1: One day of palmitate treatment, 2: Two days of palmitate treatment, 3: Three days of palmitate treatment. All data were normalized to the premenopausal control group. Data are presented as means ± SEM.

After three days of palmitate treatment the phosphorylation of JNK was higher in post-myotubes than in pre-myotubes (22% (CI 95%: 4%; 34%) vs. −12% (CI 95: −26%; 2%), (p = 0.007)), *figure 2a, c*. The expression of Hsp70 was increased after two (94% (CI 95%: 33%; 155% vs. −10% (CI 95%: −50%; 28%), p = 0.03) and three days (114% (CI 95: 50%; 177%) vs. 7% (CI 95: −78%; 91%), p = 0.04) of palmitate treatment in post-myotubes but not in pre-myotubes, *figure 2a, d*.

Correlation analyses were run on lipid metabolites and protein expression of inflammatory parameters (p-JNK and Hsp70) to find possible associations.

Increased ceramide levels in the myotubes were associated with increased Hsp70 protein expression (r = 0.31, p = 0.04). This association was driven by a correlation in the post-myotubes (r = 0.52, p = 0.02), whereas there was no significant correlation between ceramide levels and Hsp70 expression in the pre-myotubes (r = −0.16, p = 0.44). Also TAG- (r = 0.31, p = 0.04) and DAG (r = 0.30, p<0.05) levels were correlated to Hsp70 expression, which was also a reflection of an association only in the postmenopausal group (TAG (r = 0.64, p = 0.002) and DAG (r = 0.73, p = 0.0003)).

There was no association between lipid metabolites and pJNK protein expression (r = 0.13, p = 0.37).

Neither palmitate treatment, nor menopausal status affected protein expression of IκBα in the myotubes, *figure 2a, f*.

MyHC expression was not affected by either palmitate treatment or menopausal status, *figure 2*.

### Palmitate treatment and insulin sensitivity

**Figure 3:**
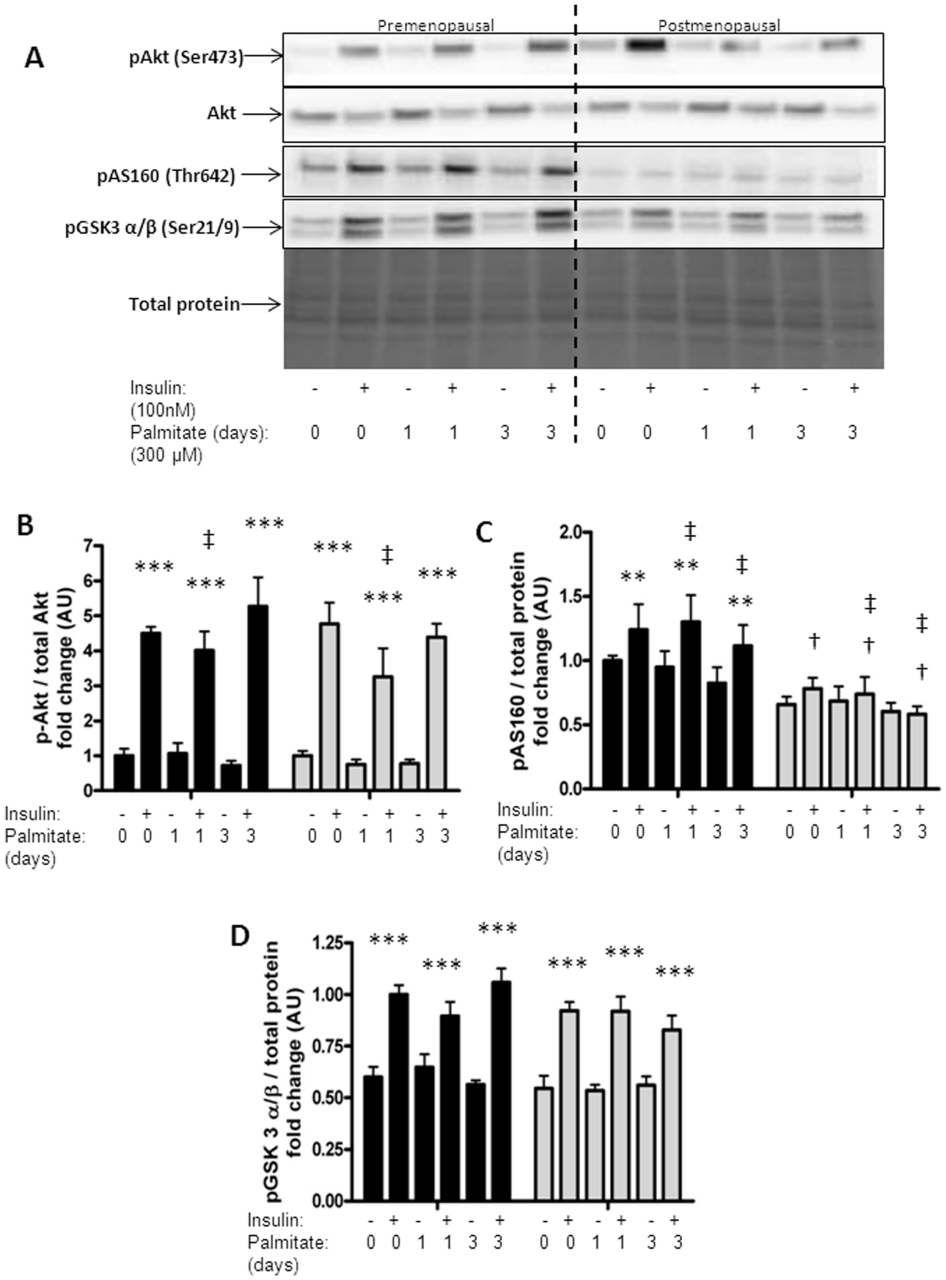
Protein expression in response to insulin in palmitate treated myotubes from premenopausal and postmenopausal women. Satellite cells were isolated from vastus lateralis biopsies from premenopausal and postmenopausal women. Cells were grown in culture until mature myotubes were formed. Myotubes were treated with palmitate (300 µM) in the last one or three days of differentiation as well as insulin (100 nM) for the last 15 minutes prior to the harvest. Protein lysates from the myotubes were immunoblottet to assess the protein expression of specific proteins as stated below. Protein expression in pre-myotubes (n = 6, black bars) and post-myotubes (n = 5, white bars, (A) representative blots of investigated proteins, (B) p-Akt-Ser473 (60 kDa) increased significantly in response to 15 minutes of insulin stimulation (100 nM). Furthermore, one day of palmitate treatment led to a significant decrease in insulin stimulated phosphorylation of Akt in the myotubes from all the women, (C) Palmitate treatment decreased insulin stimulated p-AS160-Thr642 (160 kDa) in both pre-myotubes and post-myotubes. Insulin stimulation had less of an effect on phosphorylation of AS160 in post-myotubes compared to pre-myotubes (insulin*menopause interaction), (D) p-GSK3 α/β-Ser21/9 (51/46 kDa) increased significantly in response to 15 minutes of insulin stimulation (100 nM) in both groups. Significant effect of insulin; ** p<0.01, *** p<0.0001. Significant effect of palmitate treatment; ‡ p<0.05. Significantly different from premenopausal; † p<0.05. 0: Zero days of palmitate treatment (control), 1: One day of palmitate treatment, 2: Two days of palmitate treatment, 3: Three days of palmitate treatment. All data were normalized to the premenopausal control group. Data are presented as means ± SEM.

To investigate whether the altered lipid profile following palmitate treatment of the post-myotubes was sufficient to affect insulin signaling we stimulated the palmitate treated myotubes with 100 nM insulin for 15 minutes. One day of palmitate treatment led to an overall significant decrease in phosphorylation of Akt in all the myotubes (p = 0.03), *figure 3a, b*. This was primarily driven by the decrease in the post-myotubes. Thus, there was a trend (p = 0.08) for a greater decrease in phosphorylation of Akt after one day of palmitate treatment in the post-myotubes as compared to the pre-myotubes (−40% (CI 95%: −68%; 12%) vs. −5% (CI 95%: −50%; 39%)).

There was no association between phosphorylation of Akt and mRNA expression of SPT1.

Palmitate treatment had an overall effect on insulin stimulated phosphorylation of AS160, leading to decreased phosphorylation of AS160 with palmitate treatment (p = 0.02), *figure 3a, c*. Insulin stimulation had less of an effect on phosphorylation of AS160 in post-myotubes compared to pre-myotubes (insulin*menopause interaction, p = 0.02). Neither palmitate treatment nor menopausal status affected GSK3 α/β phosphorylation in the myotubes, *figure 3a, d*.

### Fat metabolism following palmitate treatment

**Figure 4:**
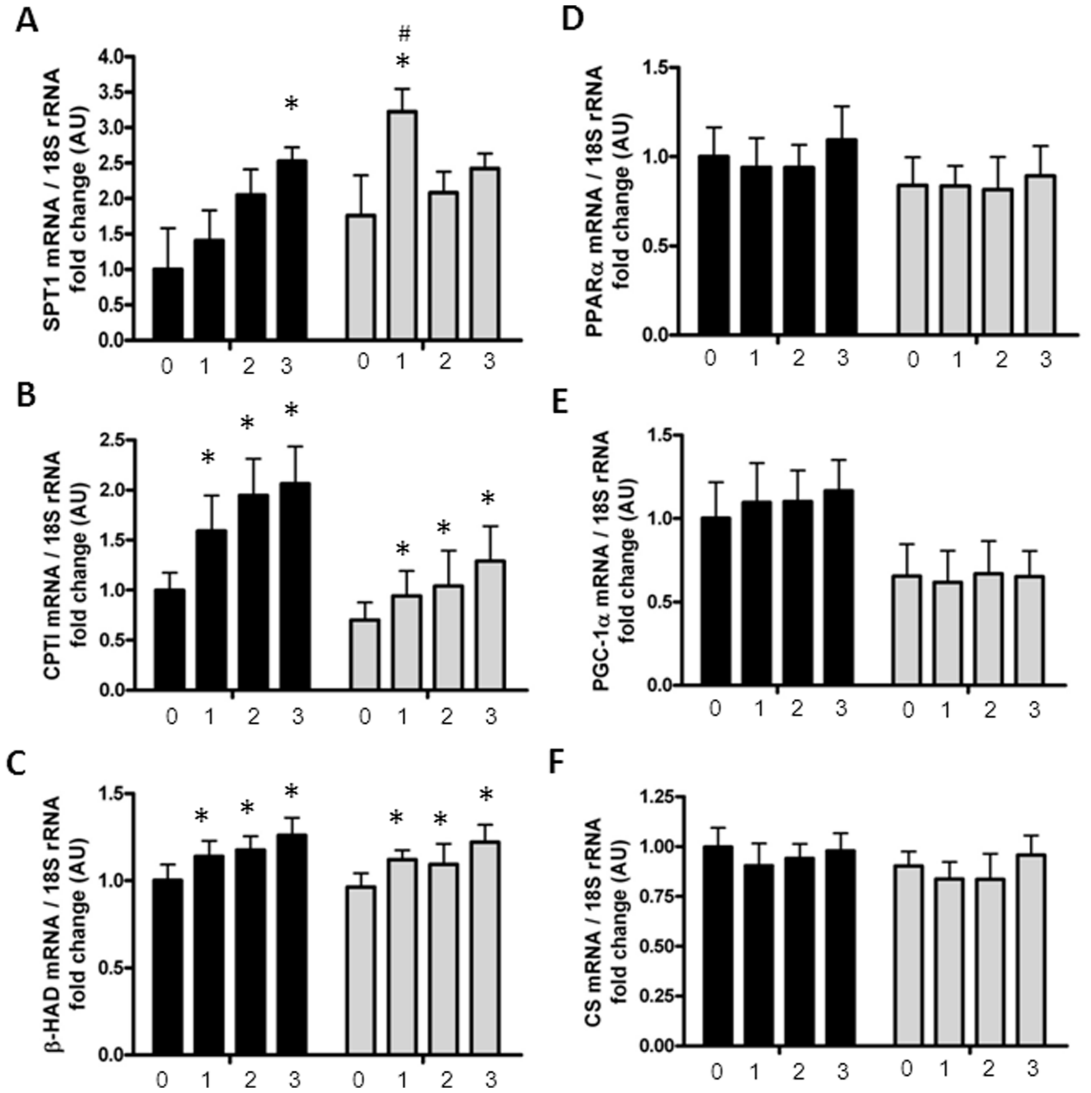
mRNA expression in palmitate treated myotubes from premenopausal and postmenopausal women. Satellite cells were isolated from vastus lateralis biopsies from premenopausal and postmenopausal women. Cells were grown in culture until mature myotubes were formed. Myotubes were treated with palmitate (300 µM) in the last one, two or three days of differentiation. The mRNA levels of analyzed genes and the endogenous control 18S were determined by quantitative real time PCR. mRNA expression in pre-myotubes (n = 6, black bars) and post-myotubes (n = 5, white bars) following zero-, one-, two- or three days of palmitate treatment (300 µM), (A) There was a menopause*palmitate interaction on SPT1, as one day of palmitate treatment led to a significant increase in SPT1 mRNA in post-myotubes. Furthermore, one day of palmitate treatment led to a significantly higher expression of SPT1 mRNA in post-myotubes compared to pre-myotubes. Three days of palmitate treatment increased SPT1 expression significantly in pre-myotubes, compared to the control, (B) CPTI mRNA expression increased significantly in response to palmitate treatment in myotubes from both groups, (C) β-HAD mRNA expression increased significantly in response to palmitate treatment in myotubes from both groups, (D) PPARα mRNA expression was unaffected by both palmitate treatment and menopausal status, (E) PGC-1α mRNA expression was unaffected by both palmitate treatment and menopausal status, (F) CS mRNA expression was unaffected by both palmitate treatment and menopausal status, 18S was used as the house keeping gene and did not differ between groups. 0: Zero days of palmitate treatment (control), 1: One day of palmitate treatment, 2: Two days of palmitate treatment, 3: Three days of palmitate treatment. Significantly different from control; * p<0.05. Significantly different from premenopausal, at same palmitate exposure time; # p<0.05. All data were normalized to the premenopausal control group. Data are presented as means ± SEM.

An explanation for the differences in intramyocellular ceramide content could be differences in the *de novo* synthesis of ceramides. To assess the amount of SPT in the myotubes, the mRNA expression of the SPT-subunit, SPT1 was measured, revealing a significant interaction between palmitate treatment and menopausal status (menopause*palmitate, p = 0.01), *figure 4a*. This was reflected by a significant increase in mRNA expression of SPT1 after one day of palmitate treatment in post-myotubes (p<0.05). Furthermore, one day of palmitate treatment led to a significantly higher mRNA expression of SPT1 in post-myotubes compared to pre-myotubes (p = 0.03). Three days of palmitate treatment led to a significant increase in SPT1 mRNA expression in pre-myotubes (p = 0.04). There was no difference in basal levels of SPT1 in the myotubes (p = 0.82).

Furthermore, ceramide accumulation in the myotubes was correlated to the mRNA expression of SPT1 (r = 0.38, p = 0.02).

The accumulation of ceramides in the post-myotubes could also be due to a defect in fatty acid oxidation. Therefore, we measured the phosphorylation of ACC, which is an indicator of changes in the fatty acid oxidation pathway. Surprisingly three days of palmitate treatment led to an increase in phosphorylation of ACC in post-myotubes (p = 0.007) compared to the pre-myotubes, *figure 2a, b*. βHAD mRNA expression increased with palmitate treatment (p<0.0001), but was unaffected by menopausal status, *figure 4c*.

Differences in accumulation of lipids could also be explained by differences in transportation molecules across the membranes. There was a trend (p = 0.07) for a smaller increase in CPTI mRNA expression in post-myotubes compared to pre-myotubes, *figure 4b*. There were no associations between CPTI and βHAD mRNA expression and levels of intramyocellular ceramides (data not shown). CD36 protein expression in the myotubes was unaffected by both palmitate treatment and menopausal status, *figure 2f*.

To assess potential changes in mitochondrial content between the groups in response to palmitate treatment, we measured the mRNA expression of PGC-1α, PPARα and CS, *table 2*. There were no differences between menopausal groups in the mRNA expression of the mentioned genes, nor were there any effects of palmitate treatment, *figure 4*.

**Table 2:** Primer sequences used for real time-PCR.

Gene		Primer Sequence
18S	Forward	5′- GCA ATT ATT CC CAT GAA CG -3′
	Reverse	5′-GGC CTC ACT AAA CCA TCC AA -3′
β-HAD	Forward	5′- GGC TTA GTG GCT GCG TGT -3′
	Reverse	5′- ATA AGC TTC CAC TAT CAT AGC ATG -3′
CPTI	Forward	5′- GAG TGA CTG GTG GGA AGA GTA CA -3′
	Reverse	5′- CTT GAT GAG CAC AAG GTC CA -3′
CS	Forward	5′- GCA TCT TGT CTT GTT CTT GCA -3′
	Reverse	5′- TGG CCT GCT CCT TAG GTA TC -3′
PPARα	Forward	5′- GCA CTG GAA CTG GAT GAC AG -3′
	Reverse	5′- TTT AGA AGG CCA GGA CGA TCT -3′
PGC-1α	Forward	5′- CAAGCCAAACCAACAACTTTATCTCT -3′
	Reverse	5′- CACACTTAAGGTGCGTTCAATAGTC -3′
	Probe	5′- AGTCACCAAATGACCCCAAGGGTTCC -3′
SPT1	Forward	5′- AGGAGTCACTGAACACTATG -3′
	Reverse	5′- AGCTCTCTCCAGTTCTTCCT -3′

## Discussion

The novel findings of this study were that chronic palmitate treatment of post-myotubes led to ceramide accumulation, increased inflammation and reduced insulin signaling, which was not observed in pre-myotubes. These findings suggest that postmenopausal women are more prone to accumulate ceramides in skeletal muscle in response to a high lipid load. This could have an impact on postmenopausal women's greater propensity to develop chronic inflammation and insulin resistance.

Several studies show that lipid excess may lead to an accumulation of intramyocellular lipid metabolites, coinciding with an impaired insulin response [13], [15], [38], [39]. Ceramide has been suggested to be an attractive candidate as a primary culprit in this lipid-mediated skeletal muscle insulin resistance [13], [15], [18], [38], [40]. However, the accumulations of intramyocellular lipids and insulin resistance have to our knowledge never been studied in relation to menopause.

The toxic effects of ceramides are known to work through phosphorylation of JNK [25]. Furthermore, JNK has been found to be increased in ovariectomized animals [24]. It is therefore obvious to speculate in the significance of lipid metabolites in the development of metabolic deterioration with menopause. We showed that palmitate treatment of post-myotubes led to an increase in intramyocellular content of ceramides and in phosphorylation of JNK, not seen in pre-myotubes. At the same time, we found no changes in the NFκB-pathway, which is also known to affect insulin signaling [41]. This indicates that the activation of JNK is the main mechanism leading to increased inflammation and affected insulin signaling after chronic palmitate treatment of post-myotubes.

Heat shock proteins are markers of oxidative stress that is known to lead to insulin resistance. Reduced expression of hsp72, the most inducible member of the hsp70 family, correlates with increased insulin resistance in patients with type 2 diabetes [42]. In our study, we found no differences in basal protein expression of Hsp70 between groups, corresponding with the fact that there were no differences in basal levels of insulin signaling proteins. However, palmitate treatment led to significant increases in Hsp70 in post-myotubes and was associated with the intramyocellular lipid levels. Studies have shown that Hsp72 is able to prevent lipid-induced insulin resistance [28]. It has been hypothesized that the underlying mechanism is hsp72's ability to decrease the phosphorylation of JNK in skeletal muscle, which is normally up regulated in response to palmitate exposure [43]. In this way, the increased Hsp72 expression could prevent the increasing insulin resistance, through the prevention of JNK phosphorylation, following a chronic lipid load. However, in the present study we found a significant increase in phosphorylation of JNK in post-myotubes despite the marked increase in Hsp70. This could indicate that myotubes from postmenopausal women have a defect in the anti-inflammatory response, resulting in uncontrolled increases in phosphorylation of JNK in spite of the increased hsp70 expression. Surprisingly, we found no significant correlation between phosphorylation of JNK and any of the lipid metabolites (ceramide, TAG or DAG), as has been described earlier [44]. It is possible that the lipid exposure has to be intensified or prolonged to induce such an association.

The molecular mechanisms behind- and the tissues responsible for the increasing insulin resistance with menopause have not been clarified. However, our data suggest that accumulation of toxic lipids in skeletal muscle could play a critical role. One study [24] investigated the molecular mechanisms behind skeletal muscle insulin resistance in ovariectomized rodents and found that ovariectomy led to decreased Akt signaling in response to insulin in skeletal muscle, which was associated with increased levels of phosphorylation of JNK. Our data support the notion that the same mechanism could be responsible for the affected insulin signaling in the post-myotubes. In the present study, both menopausal status and palmitate treatment affected insulin signaling, leading to a decreased phosphorylation of AS160. AS160 regulates insulin-stimulated glucose uptake in skeletal muscle, primarily by stimulating the translocation of GLUT4 to the cell surface [45] and decreased AS160 signaling has been associated to insulin resistance [46]. The lower phosphorylation of AS160 could therefore indicate a decreased glucose uptake as a consequence of palmitate treatment in the post-myotubes. Furthermore, this is in accordance with an animal study that found a decreased GLUT4 translocation with ovariectomy in rodents [47]. Surprisingly, our study showed no significant differences in phosphorylation of Akt between the two groups of myotubes, even though there was a trend towards a lower phosphorylation of Akt after one day of palmitate treatment in post-myotubes compared to pre-myotubes. Furthermore, it is possible that menopause primarily affects insulin signaling at a level downstream from Akt.

The accumulation of lipid metabolites in ectopic tissues may occur for several reasons. Previous studies have described decreased fat oxidation [33], [48], mitochondrial dysfunction [49], changes in *de novo* synthesis [18], as well as increased [23], decreased and delocalized lipid transporting proteins [50] as possible explanations.

As the primary source of ceramide, following palmitate treatment, seems to be the *de novo* synthesis [18], we tested the mRNA expression of SPT1, the rate-limiting step of the *de novo* ceramide synthesis. The significantly higher SPT1 mRNA expression in post-myotubes observed after one day of palmitate treatment could indicate a higher SPT-activity and hereby in part explain the higher cermide levels in the post-myotubes compared to the pre-myotubes. This is in accordance with the finding that the intramyocellular ceramide levels were correlated to the mRNA expression of SPT1. Surprisingly, three days of palmitate treatment led to a significant increase in SPT1 in pre-myotubes. This could indicate that the ceramide *de novo* synthesis is induced later in the pre-myotubes.

Earlier studies found that postmenopausal women had a significantly lower whole body fatty acid oxidation at rest [10] and during exercise [9]. Thus, decreased fat oxidation could be another explanation for the increased lipid accumulation in skeletal muscle from postmenopausal women. However, the specific role of skeletal muscle in the changes of fatty acid oxidation is unknown. Surprisingly, we found that palmitate treatment of myotubes led to a significantly larger increase in phosphorylation of ACC in the post-myotubes compared to pre-myotubes. As some earlier studies have found a discrepancy between ACC activity and lipid oxidation [51], we investigated the possibility that the higher phosphorylation of ACC in the post-myotubes following palmitate treatment could be due to compensatory mechanisms. We therefore examined the mRNA expression of CPTI and found a trend for a lower increase in CPTI expression following palmitate treatment in the postmenopausal group compared to the premenopausal group. However, based on the results of this study, it is unlikely that the changes in accumulation of lipid metabolites, in the myotubes, are solely explained by changes in fatty acid oxidation and CPTI. Nor could the differences in accumulation of lipid metabolites be explained by dissimilarities in the expression of the fat transportation molecule CD36, as there were no differences in expression of this protein between the myotubes from the two groups. The mitochondrial density was assessed by measuring the mRNA levels of CS, PGC-1α and PPARα. Menopause did not affect the expression of any of these mitochondrial markers in the myotubes. However, these markers are used as indicators of the mitochondrial density, and it is therefore possible that the two groups differ in mitochondrial functioning, as has been described in ovariectomized rodents [52].

Palmitate is known to induce lipotoxicity in skeletal muscle cells, including inflammation and apoptosis [53]. In these cells the lipotoxicity is mediated through increased intramyocellular concentrations of ceramide [54]. As the ceramide levels differed between the groups, one could hypothesize that the greater lipotoxicity in post-myotubes would lead to more pronounced cell death in this group. However, by microscopy, it was impossible to characterize any differences in cell density and appearance between the two groups.

It should also be noted that the applied model was based on a single lipid treatment (saturated fatty acid). This could affect the results, as this model of palmitate treatment is known to induce ceramide accumulation, rather than TAGs and DAGs [25]. However, even though a combination with oleate would have been more physiological, oleate (a monounsaturated fatty acid) is known to counteract the metabolic effects of saturated fatty acids such as palmitate [25]. Future studies utilizing mixed fatty acid treatments would be a useful extension of the present work.

In conclusion, we found that post-myotubes showed increased ceramide accumulation in response to a chronic lipid load compared to pre-myotubes. This increase in lipid metabolites was associated with increased inflammation as well as compromised insulin signaling. The increased ceramide level in the myotubes was associated with the increased mRNA expression of SPT1 in the myotubes. However, neither the protein expression of the lipid transporting molecule CD36 nor the mRNA expression of markers for mitochondrial density (CS, PGC-1α, PPAR-α) seemed to affect the ceramide levels in the myotubes. To our surprise, P-ACC, an indicator of changes in fatty acid oxidation, was increased in the post-myotubes. However, this mechanism could be compensatory.

All in all, these findings may point to the possibility that the effects in skeletal muscle of lipid metabolites may vary between phenotypes, and that female sex hormones may have a protective effect in the lipid-mediated development of inflammation and insulin resistance. Future studies should focus on validating this hypothesis in a human model to further investigate the clinical implications of these findings.
